# Oocyte aging: looking beyond chromosome segregation errors

**DOI:** 10.1007/s10815-022-02441-z

**Published:** 2022-02-25

**Authors:** Daniela Bebbere, Giovanni Coticchio, Andrea Borini, Sergio Ledda

**Affiliations:** 1 Department of Veterinary Medicine, University of Sassari, Sassari, Italy; 2 9.Baby, Family and Fertility Center, Bologna, Italy

**Keywords:** Cytoplasmic deterioration, Reproductive aging, Gene and epigenetic dysregulation

## Abstract

The age‐associated decline in female fertility is largely ascribable to a decrease in oocyte quality. This phenomenon is multifaceted and influenced by numerous interconnected maternal and environmental factors. An increase in the rate of meiotic errors is the major cause of the decline in oocyte developmental competence. However, abnormalities in the ooplasm accumulating with age — including altered metabolism, organelle dysfunction, and aberrant gene regulation — progressively undermine oocyte quality. Stockpiling of maternal macromolecules during folliculogenesis is crucial, as oocyte competence to achieve maturation, fertilization, and the earliest phases of embryo development occur in absence of transcription. At the same time, crucial remodeling of oocyte epigenetics during oogenesis is potentially exposed to interfering factors, such as assisted reproduction technologies (ARTs) or environmental changes, whose impact may be enhanced by reproductive aging. As the effects of maternal aging on molecular mechanisms governing the function of the human oocyte remain poorly understood, studies in animal models are essential to deepen current understanding, with translational implications for human ARTs. The present mini review aims at offering an updated and consistent view of cytoplasmic alterations occurring in oocytes during aging, focusing particularly on gene and epigenetic regulation. Appreciation of these mechanisms could inspire solutions to mitigate/control the phenomenon, and thus benefit modern ARTs.

## Introduction

The negative correlation between maternal age and fertility emerged from several demographic and epidemiological studies that consistently noted an increased decline in fertility beginning as early as the middle of the third decade in women [1, 2]. The marked decline in reproductive function is due to a reduction in both quantity and quality of oocytes, leading to increased risks of infertility, miscarriages, and birth defects [3]. Studies on biomarkers of ovarian reserve in women conceiving naturally indicate that a decrease in oocyte quality, rather than quantity, is mainly responsible for the age‐associated decline in female fertility [4]. Such a notion is confirmed by the fact that in assisted reproduction technology (ART) treatments maternal age effect is abrogated if women conceive with oocytes donated from young, healthy donors [5, 6].

A prime contributor to decreased gamete quality with age is aneuploidy due to incorrect chromosome segregation during meiosis [6, 7]. In women, meiosis is exceptionally error prone, with aneuploidy increasing exponentially from mid-thirties [7]. Chromosome segregation errors may occur during meiosis I or II as a consequence of three possible events: non-disjunction (NDJ) of homologous chromosomes (in meiosis I) or sister chromatids (meiosis II), premature separation of system chromatids (PSSC), or reverse segregation (RS), as comprehensively described in [7]. Interestingly, the occurrence of PSSC and RS increases as women age, while NDJ is more frequent in oocyte of young females [8]. While multiple mechanisms may lead to these events, the major candidates include progressive loss of chromosome cohesion [9, 10], compromised spindle assembly checkpoints that control chromosome attachment errors [11–13], and defects in microtubule dynamics [14].

Meiotic errors are not the only players in oocyte aging. Several molecular processes deteriorate during aging and negatively impact on fertilization and development [15]. Consistently, numerous alterations in morphological, cellular, biochemical, molecular, and epigenetic aspects were reported in the oocytes of several mammalian species, including human, as results of the aging process [15, 16].

As oocyte maturation and early embryonic development occur in absence of transcription, the transition from maternal to embryonic control crucially relies on molecules stored during oocyte growth. Gene expression studies indicate that advanced maternal age alters the presence and activity of genes involved in several relevant processes, including cell cycle regulation, spindle formation, organelle integrity, and gene regulation, with potential downstream effects on several aspects of oocyte biology [16–18].

A considerable body of literature addressed the effects of the aging process on the increase of meiotic and mitotic errors, as well as mitochondrial dysfunctions. Therefore, the present mini review mainly focuses on cytoplasmic alterations occurring in aging human and animal oocytes, with an emphasis on gene expression and epigenetic regulation. We also discuss possible connections between cytoplasmic mechanisms underlying oocyte aging and meiotic errors, aiming at inspiring new approaches to mitigate the oocyte aging process, and thus benefit modern ARTs.

## Age-related alterations in oocyte transcriptomes

Occurring in the absence of de novo transcription, oocyte maturation, fertilization, and early embryo development rely on molecules stored during oogenesis. Oocyte growth is therefore crucial for embryo fate and represents the link between folliculogenesis itself and embryogenesis.

Advanced maternal age alters oocyte gene expression [16, 18–20]. The dynamics of transcripts synthesis and storage in the oocyte are highly regulated in response to the specific need of individual molecules during development. For instance, expression of the enzyme DNA methyltransferase a (Dnmt3a) was reported already during early stages of oocyte growth in several species [21–23], in accordance with its crucial role in establishing de novo DNA methylation patterns during oogenesis [21]. Deteriorated gene regulation may therefore affect developmental competence, with potential downstream effects on essential processes underpinning oocyte to embryo transition. Inappropriate supply of molecules may lead to metabolic or mitochondrial dysfunction, altered epigenetic regulation, oxidative stress, or increased meiotic mis-segregations, which have all been observed in poor-quality gametes of advanced age women [24, 25]. Genome-wide expression studies in human and animal models support this hypothesis, showing that several physiological pathways are impaired by altered gene expression associated with oocyte aging [19].

Hamatani and collaborators [26] compared the transcriptome of mouse metaphase II (MII) oocytes collected from young and aged mice. Using a 22 k gene microarray, they detected the expression of approximately 11,000 genes, with about the 5% of them (530) showing significant expression differences between the two age groups, irrespective of global decline in transcript abundance that generally accompanies aging. The differentially expressed genes (DEGs) were involved in several pathways including mitochondrial function, oxidative stress, chromatin structure, DNA methylation, and genome stability.

A recent work by Mishina and co-Authors [27] described the aging-associated changes in the transcriptome of mouse oocytes throughout reproductive life. Single-oocyte comprehensive RNA sequencing revealed that oocytes undergo dramatic transcriptome changes at a late reproductive stage, when aging-associated chromosome segregation errors become more frequent [27, 28]. Interestingly, calorie restriction that reportedly prevents aging-associated egg aneuploidy [29] promoted a transcriptome shift in oocytes with the up-regulation of genes involved in chromosome segregation and a parallel attenuation of aging-associated reduction of chromosomal cohesin [27]. This raises the attractive hypothesis that aging effects on oocytes can be reversed or mitigated by appropriate diet regimens and perhaps other lifestyle habits. Genome-wide expression studies on human oocytes reported similar aging effects in terms of number of DEGs and affected pathways. A microarray analysis of MII oocytes showed age-related differences in transcript abundance of 342 genes involved in cell cycle regulation, chromosome alignment, sister chromatid separation, oxidative stress, and protein ubiquitination [18]. As gene expression analysis techniques evolved, genome-wide expression studies by microarray were replaced with more comprehensive and sensitive RNA deep sequencing techniques. Regardless, two recent single-cell RNA deep sequencing studies of aged versus young human MII oocytes yielded similar results [16, 19]. They identified 357 DEGs mainly associated with transcriptional activation, oxidative stress, immune function, and catalytic activity [16] and 481 DEGs mainly involved in transcription, ubiquitination, cell cycle, oxidative phosphorylation, and oocyte meiosis [19].

In addition, single human oocyte transcriptome analysis at both germinal vesicle (GV) and MII stages revealed distinct stage-dependent pathways impacted by aging, with the transcriptomes of IVM-MII oocytes more severely affected by age than those of GV oocytes (1219 and 596 DEGs, respectively [20]). A higher susceptibility to aging of the MII transcriptome was previously observed also in the mouse [28]. The underlying cause of this phenomenon is not known; we may hypothesize that a more altered transcriptome at the MII stage is due to stochastic alterations of transcript recruitment during maturation (such as impaired transcript selection, or inappropriate timing or amount of specific mRNA recruitment).

Age-associated alterations in gene regulation already occur during the initial stages of oogenesis, as shown by RNA-seq analysis of individual mouse growing follicles from reproductively young and old mice [3]. Beyond describing early effects of aging on folliculogenesis, Duncan and co-authors [3] also reported protein metabolism dysregulation as a hallmark of reproductive aging in the mammalian oocyte, which originates during the active growth phase of folliculogenesis.

## Expression of maternal effect genes in the aging oocyte

Maternal effect genes (MEGs) are expressed in the growing oocyte. They provide essential mRNAs and proteins to guide early embryonic development before and after embryonic genome activation [30, 31]. Females carrying defective MEGs are healthy, but at risk of reproductive failure due to early developmental arrest or imprinting disorders of their offspring [31]. Recently, human genetic studies confirmed the involvement of several MEGs in reproductive disorders, including primary infertility due to preimplantation embryonic lethality [32–36]. Playing such important roles in embryo development, MEGs are potential targets of age-associated changes in aging oocytes. Indeed, altered expression of MEGs was observed both in animal models and in women of advanced reproductive age. In the mouse, the expression of two MEGs, *Nlrp5* and *Tcl1*, was significantly decreased in polar bodies and in sibling oocytes collected from aged mice compared with young subjects [37]. Tcl1 plays vital roles during the first stages of embryo development, and its absence in the mouse oocyte has a direct impact on the cleavage potential and on the progression of the embryo beyond the blastocyst stage [38]. Its altered expression in the oocyte may therefore result in abnormal embryogenesis. Nlrp5, previously referred to as Mater, is encoded by one of the earliest identified MEG [39]. It has a direct impact on cleavage potential and progression of the mouse embryo beyond the 2-cell stage [39, 40]. Several animal and human studies revealed the involvement of Nlrp5 in reproductive aging. Significant age-associated decreases in mRNA abundance were shown in oocytes of old mice [26, 41], during late stages of oogenesis in aged sheep [42] and in oocytes of women of advanced reproductive age [17]. Nlrp5 is involved in several processes relevant to mitochondrial function and genomic imprinting [43, 44], but its mode of action is not fully understood. Importantly, the encoded protein is a core member of the subcortical maternal complex (SCMC), a multiprotein complex functionally conserved across mammals essential for early embryogenesis and female fertility [45]. Since its recent discovery [45], several studies have indicated a role of the complex in key processes buttressing oocyte to embryo transition, including meiotic spindle formation and positioning, regulation of translation, organelle redistribution, and epigenetic reprogramming (reviewed in [46]). All SCMC proteins known to date are encoded by MEGs [45, 47] expressed during oocyte growth [42, 45]; nevertheless, the temporal dynamics of SCMC assembly and mutual binding properties of its components remain unclear. Although several aspects of the SCMC remain unresolved, this structure is uniquely found in the oocyte and therefore most probably affected by advanced maternal age, with important effects on oocyte developmental competence. Gene expression studies support such a hypothesis, reporting altered expression of single SCMC members in relation to reproductive aging. In sheep, expression of six SCMC components is reduced in aged oocytes compared with oocytes of adult and prepubertal donors. More specifically, expression of *KHDC3L*, *NLRP2*, *NLRP5*, *OOEP*, *PADI6*, and *TLE6* (but not *ZBED3*) significantly declines during the latest phase of oocyte growth in aged, but not in adult or prepubertal, ewes [42]. Two independent mouse studies consistently reported altered expression of *Nlrp5* in aged oocytes at the MII stage [24, 41]. A possible impact of age on the constitution of the SCMC, namely expression of its component NLRP5, was observed also in human by Reyes et al. [17], who performed a genome-wide transcription analysis in GV and MII oocytes derived from young or advanced age donors.

The expression of *Ooep*, an additional member of the SCMC, was reduced in mouse oocytes from advanced age females [48]. In addition to its function as a component of the complex, Ooep participates in homologous recombination-mediated DNA double-strand break (DSB) repair in mouse oocytes [48]; it therefore contributes to preserve oocyte genomic integrity from DNA DSB induced by apoptosis and meiosis delay. Indeed, a decline in DNA DSB repair competence is considered a major factor contributing to oocyte and ovarian aging at advanced maternal age [49–52].

Recent studies on the genetics of female infertility identified several mutations in MEGs that cause severe reproductive disorders [32–36]. These findings underline the crucial role of such genes during early embryo development. The reported deterioration of synthesis or storage of MEG-encoded proteins in aged oocytes is likely to have severe effects on oocyte developmental competence. Equally, a functional impairment of the SCMC per se during oocyte aging is likely to affect oocyte potential [32–36, 53].

## Do cytoplasmic alterations cause meiotic errors?

The lack of Nlrp5 in mouse oocytes does not block meiosis initiation and completion but causes severe centromere cohesion weakening [41]. Such phenotype closely mirrors that observed in natural aging mouse models [9, 10], whose underlying mechanisms remain largely unknown. Centromere cohesion weakening predisposes to precocious sister chromatid separation, misalignment of chromosomes in metaphases I (MI) and MII, and high aneuploidy incidence [41]. Therefore, lack of proper amount of Nlrp5, and potentially of the SCMC in toto, may cause loss of cohesion observed in oocytes prone to meiotic errors [8–10], including oocytes of aged donors.

These studies suggest the intriguing hypothesis that meiotic errors and cytoplasmic alterations may be tightly associated; more specifically, meiotic errors may be caused or facilitated by the cytoplasmic alterations repeatedly reported in oocytes of aged donors. In accordance with this hypothesis, the comparison of transcriptomes between normal and aneuploid human embryos identified 327 deregulated DEGs, some of which produce proteins involved in spindle assembly and chromosome alignment [54].

**Fig. 1:**
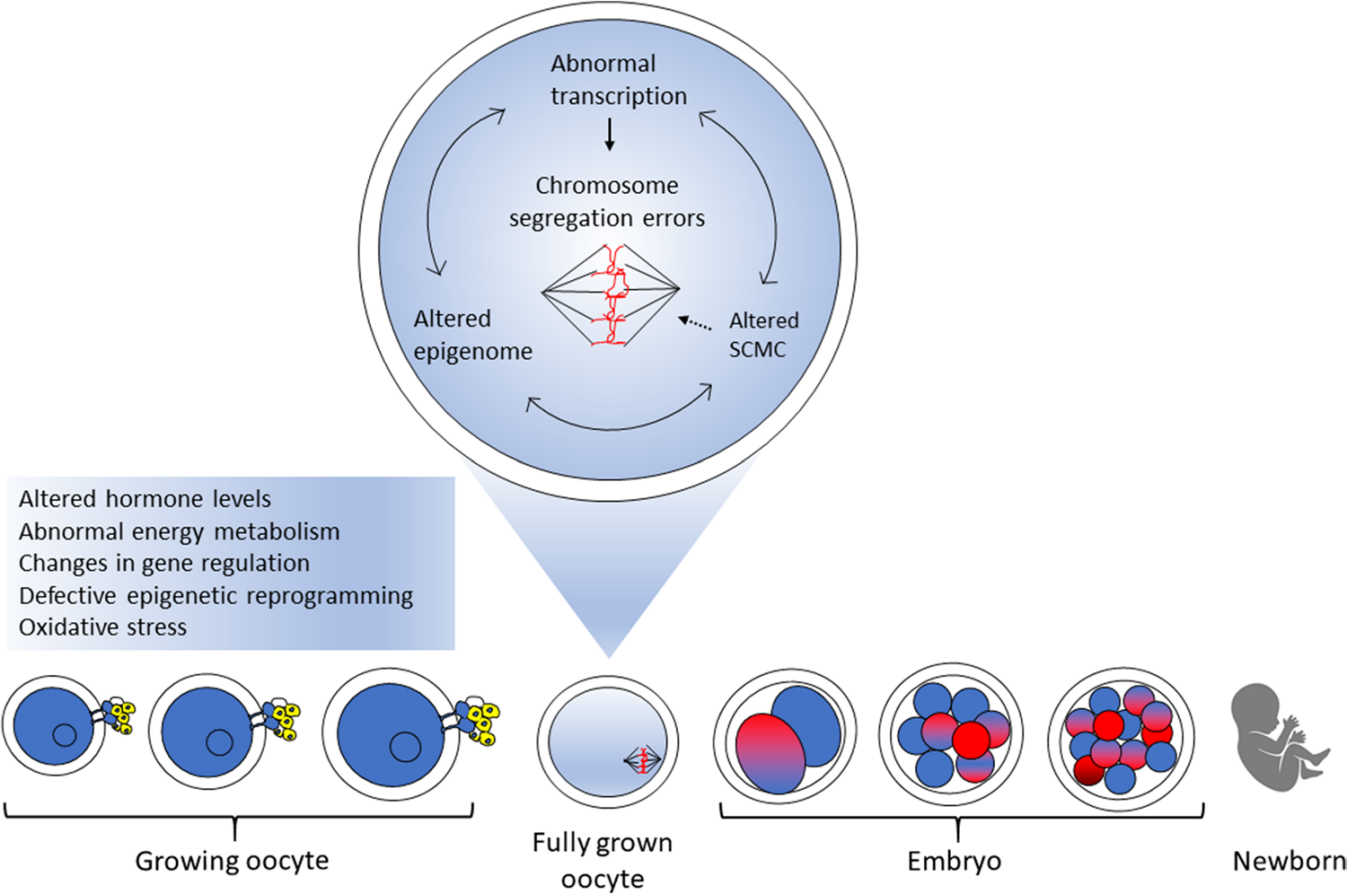
Cytoplasmic deterioration of the aging oocyte. Exposure of the growing oocyte to age-associated changes in the ovary may induce alterations in gene and epigenetic regulation, with possible effects on the subcortical maternal complex (SCMC) assembly, spindle formation, and chromosome segregation

Defects in the cytoskeletal components may also induce meiotic non-disjunction occurring with advanced reproductive age [14, 55]. Reciprocal nucleus transfer between young and aged mouse oocytes allowed to investigate the impact of age on nuclear-cytoplasmic interactions. Multipolar spindles formed in the majority of oocytes carrying “young” nuclei and “old” cytoplasm, while “young” cytoplasm rescued the age effect on spindle formation in oocytes with nuclei from “aged” oocytes. This indicates that multipolar spindles that contribute to oocyte aneuploidy are indeed a feature of aging inherently associated with microtubules, but not with chromosomes [14]. An association between cytoplasmic alterations and meiotic errors was previously demonstrated by a study on *TUBB8*, which encodes a human- and primate-specific isoform of beta tubulin [56]. Uniquely expressed in the developing oocyte, TUBB8 accounts for almost all the expressed β-tubulin and provides an essential component of the oocyte spindle; mutations in *TUBB8* gene impede meiotic spindle assembly and are responsible for human oocyte meiosis I arrest [56]. These studies strongly support a connection between age-driven cytoplasmic deterioration and the high predisposition of aged oocytes to chromosome errors. The underlying molecular mechanisms are emerging and include inadequate supply of molecules involved in meiosis, cytoskeleton modifications, or energy production. Altered expression or function of microtubules-organizing proteins and motors may disrupt normal spindle assembly [28], while alterations in the SCMC may affect proper spindle positioning and cytoskeletal actin [46, 57]. In addition, mitochondria dysfunction may cause a reduction in spindle ATP supply, as repeatedly reported during reproductive aging [58] (Fig. 1).

## Age-related alterations of oocyte epigenetics

Together with transcriptional alterations, epigenetic changes, especially DNA methylation, may contribute to compromised oocyte quality with advanced maternal age [25, 59, 60]. The changing environment of the aging ovary, such as altered hormone levels, and changes in energy and one-carbon metabolism, could affect oocyte gene epigenetic regulation [59, 61]. Indeed, transcriptome deterioration may affect the supply of key molecules involved in epigenetic processes, leading to defective epigenetic reprogramming during oogenesis and/or early embryo development.

Genome-wide DNA methylation reprogramming occurs twice during the life on an individual: during gametogenesis and in pre-implantation embryos [62]. The first extensive demethylation event starts in primordial germ cells, where the pre-existing somatic epigenetic profile is erased. Germ cell– and sex-specific epigenetic patterns are then established during later phases of gametogenesis [63]. The second event of genome-wide epigenetic reprogramming occurs in the embryo starting from fertilization. Such remodeling is required to start the new developmental program of the nascent embryo.

De novo methylation is an intricately regulated process mediated by the Dnmt3a and Dnmt3b enzymes [64], which are extensively involved in re-methylation during oogenesis [65] and embryo development [66]. The process may be influenced both by intrinsic and extrinsic factors, including changes induced by advanced age. Altered abundance of *Dnmt3b* was reported in aged mouse oocytes [26]. Dnmt3b alteration may potentially hinder proper DNA methylation establishment, including modifications of imprinted genes, with possible downstream consequences on oocyte developmental potential and embryo methylome. Decreased expression of *Dnmt3a* and *Dnmt3l* as well as the maintenance methyltransferase *Dnmt1* also occurs in aging mouse oocytes, both at the transcript and protein level [26, 67]. Direct evidence of changes in the methylome of aged oocytes was reported again in mouse. Parallel, single-cell assessment of the transcriptome and DNA methylome of fully grown GV oocytes from female mice of different age showed a lower average methylation level in oocytes from older females, together with a higher heterogeneity in gene expression [61]. Consistent results were obtained in a similar model of young and old mice, which showed age-dependent decreased expression of *Dnmt1*, *Dnmt3a*, *Dnmt3b*, and *Dnmt3L* in MII oocytes, together with lower genome-wide DNA methylation in MII oocytes and embryos [67]. Crucially, changes in the oocyte epigenetic status are also important because they can be potentially inherited by the embryo, emerging in the fully formed organism [61].

Limited information is available on the epigenetic status of the human aging oocyte. Battaglia and collaborators [68] showed altered expression of twelve microRNAs (miRNAs) in oocytes of women of advanced reproductive age. MiRNAs are post-transcriptional regulators that modulate translation and stability of mRNA targets by binding to their 3′ untranslated region [69]. In reproduction, they are important regulators of oogenesis [70–72], spermatogenesis [73–75], and early embryogenesis [76, 77]. They also play a role in the crosstalk between the oocyte and associated granulosa cells [78]. Dell’Aversana and collaborators [79] investigated the impact of aging on miRNA expression in cumulus oocyte complexes of women of different age receiving the same gonadotropin treatment during an ART cycle. Different miRNA expression profiles were observed in correlation with age, with higher levels and diversity of miRNAs detected in younger patients. Overall, these findings suggest that that oocyte aging also affects miRNA expression.

In the human, evidence of an impact of age on oocyte DNA methylation is lacking. Lower expression of the *TP73* gene in MII oocytes of aged patients [80] is consistent with this hypothesis. *TP73* is a maternally expressed imprinted gene that encodes a member of the p53 transcription factor family involved in maintenance of germ-line genomic integrity [81, 82]. As its promoter is regulated by differential DNA methylation [83], altered DNA methylation can indeed influence its expression. Nevertheless, the methylome of the aging oocyte remains a poorly explored field.

## Conclusions

Abundant evidence in human and animal models currently indicates that the decrease in oocyte developmental competence during reproductive aging is associated with significant alterations of cytoplasmic processes, in particular abnormal gene expression and epigenetic dysregulation. As our comprehension of the underlying molecular mechanisms increases, the connection between cytoplasmic deterioration and increased rates of aneuploidies becomes more evident. Such awareness will lead to novel perspectives for future research and possibly new strategies of intervention aimed at reducing the impact of age on female reproduction. As the progressive decline in cytoplasm quality is strongly linked to gene regulation during the entire period of oocyte growth, such aspect should be considered by future strategies, both in terms of novel research approaches and interventions to mitigate aging effects (i.e., lifestyle modification or nutritional interventions). Advances in understanding the phenomenon will also benefit several aspects of reproductive medicine, ranging from early recurrent embryo loss to genetics of infertility.
